# Supplementary material for: Associations between spice or pepper (Capsicum annuum) consumption and diabetes or metabolic syndrome incidence
Source: PLoS One. 2025 Feb 11;20(2):e0314448. doi: 10.1371/journal.pone.0314448 (PMC11813124; doi:10.1371/journal.pone.0314448)
Supplement: S1 Table — (DOCX) [file pone.0314448.s001.docx]

| **Variables** | **Quartiles of Spices** | | | | |
| --- | --- | --- | --- | --- | --- |
|  | **Q1** | **Q2** | **Q3** | **Q4** | **P-value** |
| **Spice (g/day)** | 0.25±0.16 | 1.0±0.30 | 1.9±0.31 | 3.4±1.7 |  |
| **Baseline age (years)** | 39.4±13.8 | 36.3±12.1 | 35.9±12.0 | 35.5±12.3 | <0.001 |
| **Sex (% women)** | 489 (44.9%) | 599 (55%) | 728 (66.6%) | 843 (77.9%) | <0.001 |
| **smoking:** |  | | | | |
| **Never + past** | 953 (87.6%) | 966 (86.6%) | 1000 (91.5%) | 1005 (92.9%) | <0.001 |
| **Current smoker** | 135 (12.4%) | 124 (11.4%) | 93 (8.5%) | 77 (7.1%) |  |
| **Physical activity (MET/min/week)** | 2269±3752 | 2134±3332 | 2164±3144 | 2178±3039 | 0.802 |
| **BMI (kg/m2)** | 25.8±4.3 | 25.8±4.2 | 25.7±4.2 | 25.8±4.4 | 0.826 |
| **Waist circumference (cm)** | 88±11.1 | 87.3±11.3 | 85.9±11.5 | 85.6±11.6 | <0.001 |
| **SBP (mm Hg)** | 100.58±14.2 | 107.78±13.2 | 106.70±12.8 | 106.28±12.9 | <0.001 |
| **DBP (mm Hg)** | 73.0±9.7 | 72.1±9.4 | 71.2±9.1 | 71.4±9.5 | <0.001 |
| **Total cholesterol (mg/dl)** | 181.32±36.8 | 179.99±35.9 | 178.24±34.5 | 178.89±37.1 | 0.208 |
| **TG (mg/dl)** | 115.70±61.5 | 113.87±56.7 | 108.32±56.5 | 106.63±55.1 | <0.001 |
| **HDL-c (mg/dl)** | 47.0±10.5 | 47.6±11.4 | 48.7±10.9 | 49.7±11 | <0.001 |
| **FBG (mg/dl)** | 90.4±14.8 | 89.8±14.7 | 89.3±12.4 | 88.7±12.3 | 0.035 |
| **Education:** |  | | | | |
| **Elementary** | 128 (11.8%) | 96 (8.8%) | 90 (8.2%) | 104 (9.6%) | 0.020 |
| **Diploma** | 643 (59.1%) | 649 (59.5%) | 679 (62.1%) | 686 (63.4%) |  |
| **Higher diploma** | 317 (29.1%) | 345(31.7%) | 324 (29.6%) | 292 (27%) |  |

**Table S1: Baseline characteristics of adult participants across quartiles of spice intake: the Tehran Lipid and Glucose Study (Mets).**

Values are Mean ± SD unless otherwise listed. P values were derived from analysis of variance and Chi-square test for continuous and dichotomous variables, respectively.
