# Supplementary material for: Associations between spice or pepper (Capsicum annuum) consumption and diabetes or metabolic syndrome incidence
Source: PLoS One. 2025 Feb 11;20(2):e0314448. doi: 10.1371/journal.pone.0314448 (PMC11813124; doi:10.1371/journal.pone.0314448)
Supplement: S2 Table — (DOCX) [file pone.0314448.s002.docx]

**Table S2. Dietary intakes across quartiles of spice intake (Mets).**

| **Quartiles of spices** | | | | | **Variables** |
| --- | --- | --- | --- | --- | --- |
| **P-value** | **Q4** | **Q3** | **Q2** | **Q1** |  |
|  | **3.4±1.7** | **1.9±0.31** | **1.0±0.30** | **0.25±0.16** | **Spices (g/day)** |
| <0.001 | 57.6 ± 5.61 | 58.5± 4.98 | 59.0 ± 5.34 | 59.7 ± 5.75 | **Carbohydrate (% of energy)** |
| 0.005 | 14.6 ± 2.5 | 14.7 ± 2.4 | 14.7 ± 2.2 | 14.9 ± 3.5 | **Protein (% of energy)** |
| <0.001 | 31.4 ± 5.20 | 30.4 ± 4.69 | 29.5± 4.98 | 28.8± 7.42 | **Total fat (% of energy)** |
| 0.03 | 10.0 ± 2.45 | 9.81 ± 2.13 | 9.58 ± 2.05 | 9.81 ± 6.88 | **SFA (% of energy)** |
| <0.001 | 6.40 ± 1.67 | 6.18± 1.51 | 5.97 ± 1.63 | 5.89 ± 5.96 | **PUFA (% of energy)** |
| <0.001 | 10.6 ± 2.28 | 10.2 ± 1.86 | 9.89 ± 2.20 | 9.80 ± 6.12 | **MUFA (% of energy)** |
| <0.001 | 11.1±5.42 | 10.8±3.36 | 10.2±3.11 | 10.2±3.35 | **Fiber (g/1000 kcal)** |
| <0.001 | 2453 ± 722 | 2410 ± 697 | 2337± 707 | 2213 ± 709 | **Energy intake, kcal/d** |

Values are Mean ± SD. ANOVA test; P for trend, across percentile spice group were performed by assigning continuous variables in a liner regression model.
