# Supplementary material for: Associations between spice or pepper (Capsicum annuum) consumption and diabetes or metabolic syndrome incidence
Source: PLoS One. 2025 Feb 11;20(2):e0314448. doi: 10.1371/journal.pone.0314448 (PMC11813124; doi:10.1371/journal.pone.0314448)
Supplement: Attachment — Submitted filename: PONE-D-24-24401_reviewer.pdf [file pone.0314448.s003.pdf]

PLOS ONE

Associations between Spice or Pepper (Capsicum annuum) Consumption and Diabetes or Metabolic Syndrome Incidence

--Manuscript Draft--

**Manuscript Number:**	PONE-D-24-24401
**Article Type:**	Research Article
**Full Title:**	Associations between Spice or Pepper (Capsicum annuum) Consumption and Diabetes or Metabolic Syndrome Incidence
**Short Title:**	Spice or Pepper Consumption and Diabetes or Metabolic Syndrome Incidence
**Corresponding Author:**	Firoozeh Hosseini-Esfahani, Ph.D<br>Research Institute for Endocrine Sciences<br>Tehran, IRAN, ISLAMIC REPUBLIC OF
**Keywords:**	Type 2 diabetes; metabolic syndrome; Incidence; Spice; Capsicum; Polyphenol; Pepper
**Abstract:**	Background<br><br>Spice and pepper contain polyphenols which have beneficial effects on various aspects of health. This study aimed to investigate the association between spice or pepper consumption and the incidence of metabolic syndrome (MetS), its components or type 2 diabetes (T2D).<br><br>Methods<br><br>We included eligible adults with a median follow-up of 5.8 years among the participants of the Tehran lipid and glucose study. Baseline and follow-up examinations were conducted to assess dietary, biochemical and anthropometric variables. Multivariable Cox proportional hazard regression models were used to determine the association of spice or pepper intakes and hazard ratios of T2D, MetS or its components.<br><br>Results<br><br>The analysis was performed on 2303 men and 3037 women (n=5340), with a mean age of 39.9±13.4 and 406 incident cases of T2D. Also, 4353 participants including 1694 men and 2659 women were included for MetS analysis with 1211 incident cases of MetS. After adjusting for confounding factors, spice and pepper intakes did not associate with the incidence of T2D or MetS. Further, in the upper quartile of spice intake, hazard ratio of high triglyceride (TG) increased [HR Q4: 1.19 (CI: 1.05-1.35)]. Also, spice intake increased the incidence of high blood pressure (BP) [HR: 1, 1.03 (0.93-1.14), 1.06 (0.96-1.17), 1.16 (1.04-1.30), P-trend=0.007]. The risk of low high density lipoprotein cholesterol (HDL-C) appeared to decrease in the third quartile of the pepper consumption (HR: 1, 0.97, 0.87, 1.03, P-trend=0.008).<br><br>Conclusion<br><br>Our results indicated that spice and pepper intakes had no association with incidence of T2D and MetS. In the upper quartiles of spice intake, incidence of high TG and BP elevated. Lower consumption of pepper decreased the incidence of low HDL-C.
**Order of Authors:**	Azam Ildarabadi
	Firoozeh Hosseini-Esfahani, Ph.D
	Shahrzad Daei
	Parvin Mirmiran
	Fereidoun Azizi
**Additional Information:**	

Powered by Editorial Manager® and ProduXion Manager® from Aries Systems Corporation

Question	Response
**Financial Disclosure**<br><br>Enter a financial disclosure statement that describes the sources of funding for the work included in this submission. Review the submission guidelines for detailed requirements. View published research articles from *PLOS ONE* for specific examples.<br><br>This statement is required for submission and **will appear in the published article** if the submission is accepted. Please make sure it is accurate.<br><br>**Funded studies**<br>Enter a statement with the following details:<br>• Initials of the authors who received each award<br>• Grant numbers awarded to each author<br>• The full name of each funder<br>• URL of each funder website<br>• Did the sponsors or funders play any role in the study design, data collection and analysis, decision to publish, or preparation of the manuscript?<br><br>Did you receive funding for this work?	Yes
Please add funding details.<br>as follow-up to "**Financial Disclosure**<br><br>Enter a financial disclosure statement that describes the sources of funding for the work included in this submission. Review the submission guidelines for detailed requirements. View published research articles from *PLOS ONE* for specific examples.<br><br>This statement is required for submission and **will appear in the published article** if the submission is accepted. Please make sure it is accurate.	This work was supported by the Research Institute for Endocrine Science, Shahid Beheshti University of Medical Science (Tehran, Iran) under Grant number. 43003670

Powered by Editorial Manager® and ProduXion Manager® from Aries Systems Corporation

**Funded studies**<br>Enter a statement with the following details:<br>• Initials of the authors who received each award<br>• Grant numbers awarded to each author<br>• The full name of each funder<br>• URL of each funder website<br>• Did the sponsors or funders play any role in the study design, data collection and analysis, decision to publish, or preparation of the manuscript?<br><br>Did you receive funding for this work?"	
Please select the country of your main research funder (please select carefully as in some cases this is used in fee calculation).<br>as follow-up to "**Financial Disclosure**<br><br>Enter a financial disclosure statement that describes the sources of funding for the work included in this submission. Review the submission guidelines for detailed requirements. View published research articles from *PLOS ONE* for specific examples.<br><br>This statement is required for submission and **will appear in the published article** if the submission is accepted. Please make sure it is accurate.<br><br>**Funded studies**<br>Enter a statement with the following details:<br>• Initials of the authors who received each award<br>• Grant numbers awarded to each author<br>• The full name of each funder<br>• URL of each funder website<br>• Did the sponsors or funders play any role in the study design, data collection and analysis, decision to publish, or preparation of the manuscript?<br><br>Did you receive funding for this work?"	IRAN, ISLAMIC REPUBLIC OF - IR
**Competing Interests**	The authors have declared that no competing interests exist.

Use the instructions below to enter a competing interest statement for this submission. On behalf of all authors, disclose any competing interests that could be perceived to bias this work—acknowledging all financial support and any other relevant financial or non-financial competing interests.<br><br>This statement is required for submission and **will appear in the published article** if the submission is accepted. Please make sure it is accurate and that any funding sources listed in your Funding Information later in the submission form are also declared in your Financial Disclosure statement.<br><br>View published research articles from *PLOS ONE* for specific examples.<br><br>**NO authors have competing interests**<br><br>Enter: *The authors have declared that no competing interests exist.*<br><br>**Authors with competing interests**<br><br>Enter competing interest details beginning with this statement:<br><br>*I have read the journal's policy and the authors of this manuscript have the following competing interests: [insert competing interests here]*<br><br>* typeset	
**Ethics Statement**<br><br>Enter an ethics statement for this submission. This statement is required if the study involved:<br><br>• Human participants<br>• Human specimens or tissue<br>• Vertebrate animals or cephalopods<br>• Vertebrate embryos or tissues	All participants signed the written informed consent for taking part in this study. This study was approved by the ethics committee of the Research Institute for Endocrine Sciences, Shahid Beheshti University of Medical Sciences (Tehran, Iran) [21, 22]. (IR.SBMU.ENDOCRINE.REC.1401.118) (Grant no. 43003670).<br>The dates when data were accessed for research purposes were 20/5/2023.

Powered by Editorial Manager® and ProduXion Manager® from Aries Systems Corporation

• Field research<br><br>Write "N/A" if the submission does not require an ethics statement.<br><br>General guidance is provided below. Consult the submission guidelines for detailed instructions. **Make sure that all information entered here is included in the Methods section of the manuscript.**	

**Format for specific study types**<br><br>**Human Subject Research (involving human participants and/or tissue)**<br>• Give the name of the institutional review board or ethics committee that approved the study<br>• Include the approval number and/or a statement indicating approval of this research<br>• Indicate the form of consent obtained (written/oral) or the reason that consent was not obtained (e.g. the data were analyzed anonymously)<br><br>**Animal Research (involving vertebrate animals, embryos or tissues)**<br>• Provide the name of the Institutional Animal Care and Use Committee (IACUC) or other relevant ethics board that reviewed the study protocol, and indicate whether they approved this research or granted a formal waiver of ethical approval<br>• Include an approval number if one was obtained<br>• If the study involved *non-human primates*, add *additional details* about animal welfare and steps taken to ameliorate suffering<br>• If anesthesia, euthanasia, or any kind of animal sacrifice is part of the study, include briefly which substances and/or methods were applied<br><br>**Field Research**<br><br>Include the following details if this study involves the collection of plant, animal, or other materials from a natural setting:<br>• Field permit number<br>• Name of the institution or relevant body that granted permission	
**Data Availability**<br><br>Authors are required to make all data underlying the findings described fully available, without restriction, and from the time of publication. PLOS allows rare exceptions to address legal and ethical concerns. See the PLOS Data Policy and FAQ for detailed information.	No - some restrictions will apply

Powered by Editorial Manager® and ProduXion Manager® from Aries Systems Corporation

A Data Availability Statement describing where the data can be found is required at submission. Your answers to this question constitute the Data Availability Statement and **will be published in the article**, if accepted.<br><br>**Important:** Stating 'data available on request from the author' is not sufficient. If your data are only available upon request, select 'No' for the first question and explain your exceptional situation in the text box.<br><br>Do the authors confirm that all data underlying the findings described in their manuscript are fully available without restriction?	
**Describe where the data may be found in full sentences. If you are copying our sample text, replace any instances of XXX with the appropriate details.**<br><br>• If the data are **held or will be held in a public repository**, include URLs, accession numbers or DOIs. If this information will only be available after acceptance, indicate this by ticking the box below. For example: *All XXX files are available from the XXX database (accession number(s) XXX, XXX.).*<br>• If the data are all contained **within the manuscript and/or Supporting Information files**, enter the following: *All relevant data are within the manuscript and its Supporting Information files.*<br>• If neither of these applies but you are able to provide **details of access elsewhere**, with or without limitations, please do so. For example:<br><br>*Data cannot be shared publicly because of [XXX]. Data are available from the XXX Institutional Data Access / Ethics Committee (contact via XXX) for researchers who meet the criteria for access to confidential data.*<br><br>*The data underlying the results presented in the study are available from (include the name of the third party*	The datasets generated and analyzed during the current study are not publicly available because data contains sensitive individual information and data are owned by Research Institute for Endocrine Sciences, Shahid Beheshti University of Medical Sciences. Tehran, Iran<br>Data are available from the ethics committee of the Research Institute for Endocrine Sciences, Shahid Beheshti<br>University of Medical Sciences, Tehran, Iran, whenever data request has been sent.<br>No.24, Arabi Street, Yemen Avenue, Chamran Highway<br>Fax: +98 (21)22402463<br>Postal code: 1985717413<br>Email: info@endocrine.ac.ir.

and contact information or URL).<br>• This text is appropriate if the data are owned by a third party and authors do not have permission to share the data.<br><br>* typeset	
Additional data availability information:	

Powered by Editorial Manager® and ProduXion Manager® from Aries Systems Corporation

Manuscript
Click here to access/download;Manuscript;spice and T2D 12-06-2024 (1).docx

Associations between Spice or Pepper (*Capsicum annuum*) Consumption and Diabetes or Metabolic Syndrome Incidence

Azam Ildarabadi[1¶], Firoozeh Hosseini-Esfahani[1¶], Shahrzad Daei[1], Parvin Mirmiran[1,2], and Fereidoun Azizi[3]

[1]Nutrition and Endocrine Research Center, Research Institute for Endocrine Sciences, Shahid Beheshti University of Medical Sciences, Tehran, Iran

[2]Department of Clinical Nutrition and Dietetics, Faculty of Nutrition Sciences and Food Technology, National Nutrition and Food Technology Research Institute, Shahid Beheshti University of Medical Sciences, Tehran, Iran

[3]Endocrine Research Center, Research Institute for Endocrine Sciences, Shahid Beheshti University of Medical Sciences, Tehran, Iran

[¶]These two authors contributed equally

Corresponding author:

1. Parvin Mirmiran, Prof.

[1]Nutrition and Endocrine Research Center, Research Institute for Endocrine Sciences, Shahid Beheshti University of Medical Sciences, Tehran, Iran

E-mail: parvin.mirmiran@sbmu.ac.ir

Parvin.mirmiran@gmail.com

2. Firoozeh Hosseini-Esfahani

Nutrition and Endocrine Research Center, Research Institute for Endocrine Sciences, Shahid Beheshti University of Medical Sciences, Tehran, Iran

E-mail: f.hosseini@sbmu.ac.ir

Abstract

Background: Spice and pepper contain polyphenols which have beneficial effects on various aspects of health. This study aimed to investigate the association between spice or pepper consumption and the incidence of metabolic syndrome (MetS), its components or type 2 diabetes (T2D).

Methods: We included eligible adults with a median follow-up of 5.8 years among the participants of the Tehran lipid and glucose study. Baseline and follow-up examinations were conducted to assess dietary, biochemical and anthropometric variables. Multivariable Cox proportional hazard regression models were used to determine the association of spice or pepper intakes and hazard ratios of T2D, MetS or its components.

Results: The analysis was performed on 2303 men and 3037 women (n=5340), with a mean age of 39.9±13.4 and 406 incident cases of T2D. Also, 4353 participants including 1694 men and 2659 women were included for MetS analysis with 1211 incident cases of MetS. After adjusting for confounding factors, spice and pepper intakes did not associate with the incidence of T2D or MetS. Further, in the upper quartile of spice intake, hazard ratio of high triglyceride (TG) increased [HR Q4: 1.19 (CI: 1.05-1.35)]. Also, spice intake increased the incidence of high blood pressure (BP) [HR: 1, 1.03 (0.93-1.14), 1.06 (0.96-1.17), 1.16 (1.04-1.30), P-trend=0.007]. The risk of low high density lipoprotein cholesterol (HDL-C) appeared to decrease in the third quartile of the pepper consumption (HR: 1, 0.97, 0.87, 1.03, P-trend=0.008).

Conclusion: Our results indicated that spice and pepper intakes had no association with incidence of T2D and MetS. In the upper quartiles of spice intake, incidence of high TG and BP elevated. Lower consumption of pepper decreased the incidence of low HDL-C.

Keywords: Type 2 diabetes, Metabolic syndrome, Incidence, Spice, Capsicum, Polyphenol, Pepper

Introduction:

Metabolic syndrome (MetS) as a prevalent condition can lead to chronic diseases such as type 2 diabetes (T2D), cardiovascular diseases, fatty liver disease and etc [1]. (and many more?) Various clinical manifestations such as waist circumference (WC), (WC above a certain reference value?) high blood pressure (BP), impaired fasting blood glucose level (FBG), high level of triglyceride (TG), and high density lipoprotein cholesterols (HDL-C) lower than normal levels are defined as MetS [2]. T2D represents a growing public health concern that is associated with increased morbidity and mortality [3]. Also, it seems that the prevalence of T2D and obesity coincide with frequency of MetS. While the MetS complications are increased, the management and prevention of this condition is raising interest (1). Inflammation is a serious problem in diabetic patients. Inflammation increases the levels of circulating inflammatory cytokines and insulin resistance in the liver, skeletal muscles, and vascular endothelium [4, 5]. In this regard, spice and pepper recognized as a source of antioxidants and anti-inflammatory compounds; so, their use has gained attention in diabetes research [6]. (Improve this sentence) Further, the using of herbs and spices has been paid more attention, while the modern medicine has been improved. Herbs and spices have compounds called polyphenols. Polyphenols have potential beneficial effects in the body which are related to decreasing MetS [7]. Epidemiological and randomized clinical trial studies conducted on human and animal models have provided substantial evidence that dietary intake of phenolic compounds (PC) plays a vital role in reducing the risk of diabetes. PC regulates significant pathways involved in carbohydrate metabolism and hepatic glucose homeostasis, including glycolysis, glycogenesis, and gluconeogenesis [8, 9]. Furthermore, PC has been found to have a direct inhibitory effect on the development of inflammation and can suppress appetite by inducing the secretion of

cholecystokinin (CCK) and leptin; such action of PC may have an anti-obesogenic effect and provide some level of protection against diabetes [9, 10].

In addition to anti-microbial, anti-inflammatories, and antioxidant properties, herbs and spices may have potential protective effects against cardiovascular disease, neurodegeneration, T2D, and cancer [11-13]. They are also known to play an important role in stabilizing lipid peroxidation and inhibiting various types of oxidizing enzymes [14]. Spices and culinary herbs also possess a high anti-glycant potential arising mainly from their polyphenol content [15].

Previous epidemiological studies reported that dietary polyphenols were associated with reduced incidence of diabetes, but these associations were controversial [16, 17]. Complementary medicine as an alternative therapy is used in various countries. Herbs and spices are considered to prevent and manage MetS due to the lack of side effects [18]; therefore, in this cohort study, the associations between spice or pepper intakes and the incidence of T2D, MetS or its components in adults were investigated.

Materials and methods:

Study populations:

Participants were selected from the Tehran Lipid and Glucose Study (TLGS), a large-scale cohort study conducted in Tehran, the capital of Iran, to identify non-communicable disease risk factors. The first examination survey was conducted from 1999 to 2001 on 15005 individuals aged ≥3 years, using the composite stratified cluster random sampling technique. This cohort study supervised follow-up examinations every 3 years; 2002–2005 (survey 2), 2005–2008 (survey 3), 2008–2011 (survey 4), 2012–2015 (survey 5), and 2015–2018 (survey 6) to identify recently developed diseases.

Of individuals participating in survey 4, 6882 subjects aged ≥18 years were? randomly selected for dietary assessment. After excluding subjects with under- or over-reporting of energy intake (<800 or ≥4,200 kcal/day) (n=470) [19], 6412 adult men and women remained as baseline participants and followed up until survey 6. Of these participants, we excluded pregnant or lactating women and subjects diagnosed with T2D based on measurements of FBG or self-reported taking glucose-lowering drugs (n=523). Also, subjects without biochemical and anthropometric data (n=84) were excluded. After omitting participants missing any follow-up data (n=465), 5340 subjects remained and were entered for T2D secondary analysis (Figure 1).

Figure 1. Outline of study participants

Subjects participating in survey 4 (baseline), aged ≥18 years, and completed the dietary assessment entered in this study for MetS secondary analysis. Subjects with MetS diagnosis at baseline [20], pregnant or lactating women were excluded. Also, individuals with over- or under-reporting of energy intake (≥4,200 or <800 kcal/day) were omitted [19]. A total of 4517 adult women and men with accessible biochemical, anthropometric and dietary data were selected as the baseline population and they tracked until survey 6. Subjects who did not provide follow-up data removed from analysis, as a result, 4353 subjects remained and pass into the analysis. Other independent lines of exclusion were done for MetS components, including abdominal obesity, high BP, low HDL-C, high FBG and high TG (Figure 2).

Fig 2. Outline of study participants' selection

All participants signed the written informed consent for taking part in this study. This study was approved by the ethics committee of the Research Institute for Endocrine Sciences, Shahid Beheshti University of Medical Sciences (Tehran, Iran) [21, 22]. (IR.SBMU.ENDOCRINE.REC.1401.118) (Grant no. 43003670).

The dates when data were accessed for research purposes were 20/5/2023.

Dietary intake measurements:

Dietary assessment was done by expert nutritionist using a valid and reliable 168-item semi-quantitative food frequency questionnaire (FFQ) [23, 24]. Usual dietary intakes were collected through face-to-face personal interviews based on standard portion sizes. The consumption of common spices in the diet was measured using the FFQ; also, peppers include bell peppers and green peppers. Based on the frequency consumption of each food item on a daily, weekly, monthly, or yearly basis, daily intakes were calculated. Portion sizes were then converted to mass (gr/day) using household measurements. The USDA (United States Department of Agriculture) Food Composition Table (FCT) was used to calculate energy and nutrient intakes. National foods not listed in the USDA FCT were provided with the Iranian FCT [25]. Although the nutrient contents of food items were computed cumulatively across follow-up surveys from the baseline survey until the point of the last follow-up visit or the time that diabetes, MetS or its components were diagnosed.

Physical Activity Measurements:

Physical activity was measured using a Persian-translated modifiable activity questionnaire (MAQ). The previous study reported high reliability and moderate validity of this questionnaire [26]. Time and frequency of light, middle, high, and challenging intensity activities were received as stated by routine activities of daily life over the past year. Intensity of activity (low, mild, moderate, severe), time, and frequency as a routine activity of daily life over the past year were measured. These activity data were converted into metabolic equivalent/minutes/week (MET/min/week) [27].

Blood Pressure and Anthropometric Measurements:

Body weight was measured using a digital scale (Seca 707) with an accuracy of 100 gr with light clothing. The height was measured while participants were standing in a straight position and without shoes, using a tape meter on the wall with an accuracy of 0.5 cm. A flexible measuring tape was used to determine WC and hip circumference (HC) with an accuracy of 0.1 cm. The HC was measured at its maximum over the buttocks. The WC was measured at the level of umbilicus over light clothing, at the end of a normal expiration, with an unstretched tape meter. The waist-to-hip ratio was calculated by dividing WC to HC.

BP was measured using a standard mercury sphygmomanometer based on standard protocols that were previously explained [28]. Following 15 minutes of rest, the physician fixed the cuff on the right arm of the participant and measured blood pressure while seated. The measurement was repeated twice with an interval of 30 seconds. The average of two measures was taken.

Biochemical Analysis:

The participants were asked to fast for 12-14 hours before the visit. Blood samples were collected between 7-9 am. The samples were centrifuged 30–45 min after collection. In the TLGS research laboratory, they carried out all biochemical analyses on the day of blood sample collection using the Selectra 2 auto-analyzer (Vital Scientific, Spankeren, the Netherlands). FBG was measured using enzymatic colorimetric method and glucose oxidase technique.

During follow-up, for participants who didn't use glucose-lowering drugs, the standard 2-h post-challenge blood glucose test was measured using oral administration of 82.5 g glucose monohydrate solution (equivalent to 75 g anhydrous glucose). After precipitating Apolipoprotein-B containing lipoproteins with phosphotungstic acid, the concentration of HDL-C was determined. TG level was assessed by enzymatic colorimetric tests using glycerol phosphate oxidase (Pars Azmoon Inc., Tehran, Iran). For glucose, the inter- and intra-assay

coefficients of variations were both 2.2%. For TG, inter- and intra-assay coefficients of variations were 1.6 and 0.6% [21].

Diabetes Risk Score:

To reduce the number of confounding factors in the analysis, we measured the diabetes risk score (DRS) as follows: family history of T2D (5 points) (a positive family history of diabetes was determined as having at least one parent or sibling with diabetes), systolic BP (mmHg) <120 (0 points), 120–140 (3 points), systolic BP ≥140 (7 points); TG/HDL-C: <3.5 (0 points), ≥3.5 (3 points); WC to-height ratio: <0.54 (0 points), 0.54–0.59 (6 points), ≥0.59 (11 points); FBG (mmol/L): <5.0 (0 points), 5.0–5.5 (12 points), 5.6–6.9 (33 points) [29].

Outcome Definition:

Incidence of T2D was determined as fasting plasma glucose concentrations ≥126 mg/dl or 2-h plasma glucose concentrations ≥200 mg/dl or self-reported taking glucose-lowering drugs (oral diabetes medication or insulin injections) [30]. Also, Subjects with three or more of the following criteria were diagnosed as MetS phenotype according to the Iranian modified National Cholesterol Education Program/Adult [20, 31]: (1) Abdominal obesity (WC ≥ 95 cm in men and women); (2) BP ≥ 130/85 mmHg or antihypertensive drug treatment; (3) HDL-C < 1.30 mmol/l (<50 mg/dl) in women, and <1.04 mmol/l (<40 mg/dl) in men or receiving drug treatment; (4) FBG ≥6.11 mmol/l (≥110 mg/dl) or drug treatment for hyperglycemia; (5) TG ≥1.70 mmol/l (≥150 mg/dl) or drug treatment.

Statistical Analysis:

The data were analyzed by SPSS software compatible with Windows, version 26 (IBM Inc.). A P-value of less than 0.05 is considered as significant difference. We categorized the data on spice

and pepper intakes into quartiles. The Chi-square test for qualitative variables and one-way ANOVA for quantitative variables were used to compare the mean and frequency of participants' baseline characteristics across quartiles of spice and pepper intakes. P for trends across spice and pepper intake categories were performed by assigning quantitative variables in a linear regression model.

The hazard ratios (HRs) and 95% confidence intervals (CIs) of T2D and MetS or its components incidence were estimated through multivariable Cox proportional hazard regression analyses. There were no interaction terms of spice and pepper intake with age or sex groups. The first quartile was assumed as the reference. The median of each quartile was utilized as a quantitative variable to estimate the P-value of trends across quartiles of spice and pepper intakes in the Cox proportional hazard regression models. Time to event was defined as the time between baseline and the event date (for event cases) or the last follow-up (for censored participants), whichever occurred first. The event date was defined as the mid-time between the follow-up visit date at which T2D, MetS or its components was detected for the first time and the most recent follow-up visit before the diagnosis. Study participants were omitted due to death or loss to follow-up. The Cox regression models were performed with sex and age adjustments in the crude model. In the analysis for T2D incidence, sex, age, education levels (<8, 9-12 and >12 years), smoking (never smoked, past smoker, and current smoker), physical activity, diabetes risk score, total energy, fat (percentage of energy), and fiber intake (gr/1000 kcal) were considered as cofounders in the adjusted model. In the analysis for MetS incidence, age, sex, education level, physical activity, total energy, saturated fat (percentage of energy), fiber intake (gr/1000 kcal), BMI and smoking were considered as cofounders in the adjusted model. In models to estimate the HR of high BP, high TG, high FBG, and low HDL-C respectively, the continuous amount of each risk factor at

the beginning of study corresponding to each model was added to the adjustment factors. Further, non-normally distributed variables were transformed into natural log (Ln) variables to align with normal distribution before entering the statistical models. The proportional hazard assumption was confirmed by the Schoenfeld residuals test and plot of log [-log (survival)] vs. log (time) to see if they are parallel.

Results:

This study conducted on 2303 men and 3037 women (n=5340) with the mean±SD age of women 39.0±13 and men 41.1±14; there were 406 incident cases of T2D during a mean of 5.8 years of follow-up. For MetS analysis, there were 1694 men and 2659 women (n=4353), with the mean±SD age of 38.6±14.3 and 35.9±11.8 years, respectively. During 5.8 years follow-up, 1211 subjects were found with MetS incidence. The baseline characteristics and dietary intakes of participants among quartiles of spice intake are demonstrated in Tables 1 and 2 respectively. Results have shown that current smokers were higher in the upper quartile of spice intake (Table 1). Also, WC and WC to height ratio (WHR) were lower in the upper quartile of spice intake. Furthermore, with increasing spice consumption, systolic and diastolic BP had been decreased (P<0.001). Moreover, subjects in the upper quartile of spice intake had lower TG/HDL-C ratio and FBG.

Participants who were in the upper quartile of spice intake had a decrease in carbohydrate and protein intake (Table 2). With the increasing of spice consumption, there had been an increase in total fat, polyunsaturated fatty acids (PUFAs), monounsaturated fatty acids (MUFAs), saturated fatty acids (SFAs), and energy intake. Also, fiber consumption increased in the upper quartile of spice intake.

The Cox proportional HRs for T2D according to quartiles of spice and pepper intakes were shown respectively in Tables 3 and 4. Higher intake of spice was associated with 1% increase in the incidence of T2D (HR: 1, 0.99 (0.92-1.0), 1.0 (0.94-1.1), 1.1 (1.0-1.1), P for trend=0.01); however, after adjusting for confounding variables, there was no association between spice consumption and T2D incidence. Furthermore, the consumption of pepper had no association with incidence of T2D [HR: 1, 0.97 (0.90-1.0), 0.96 (0.89-1.0), 0.95 (0.88-1.0), P trend=0.8]; also this association didn't change after adjustment for confounding variables [HR: 1, 1.0 (0.94-1.1), 1.0 (0.91-1.0), 0.98 (0.89-1.0), P-trend=0.8].

In the analysis for MetS incidence, the baseline characteristics and dietary intake of participants among quartiles of spice intake are demonstrated in Tables S1 and S2, respectively. The findings showed that individuals in the upper quartile of spice intake had lower TG, FBG, WC, and diastolic BP than the lower quartiles. Also, HDL-C was higher in the upper quartile of spice intake than the lower quartiles. Total energy intake, total fat, MUFA, PUFA, and SFA intake increased in the upper quartiles of spice. Moreover, in the upper quartiles of spice consumption, protein and carbohydrate intakes decreased.

Spice intake had no association with MetS incidence in the crude and adjusted [HR: 1.01 (0.91-1.12), 1.02 (0.92-1.14), 1.05 (0.94-1.18) P trend= 0.65] models (Table 3). Pepper consumption had no association with MetS incidence in the crude and adjusted model (Table 4). Moreover, the results showed that by increasing spice intake, the hazard ratio of BP incidence increased in the crude and adjusted [HR: 1, 1.03 (0.93-1.14), 1.06 (0.96-1.17), 1.16 (1.04-1.30), P-trend=0.007] models. Also, by increasing pepper intake, the hazard ratio of low HDL-C level significantly decreased in the quartile 3 of crude model [HR: 1, 0.97 (0.87-1.09), 0.87 (0.78-0.97), 1.03 (0.92-1.14), P-trend = 0.008].

Table 1. Baseline characteristics of adult participants across quartiles of spice intake: the Tehran Lipid and Glucose Study

Variables	Q1	Q2	Q3	Q4	P-value
Baseline age (years)	42.8±14.3	39.4 ±13.0	39.2 ± 13.0	38.2±13.1	<0.001
Sex (% women)	42	51.1	60.0	74.1	<0.001
Smoking n (%)					
Non-smoker	902 (68.2)	820 (63.0)	881 (70.0)	894 (67.2)	0.01
Ex- smoker	250 (18.9)	270 (20.7)	238(18.1)	220 16.5)	
Smoker	170 (12.9)	212 (16.3)	198 (15.0)	216 (16.2)	
Physical activity (MET/min/week)	520 ± 757	538 ± 896	532 ± 811	499 ± 758	0.60
BMI (kg/m^2)	27.1± 4.63	27.0 ± 4.72	27.0 ± 4.69	27.1 ± 4.93	0.80
WC (cm)	93.0 ± 11.7	91.8 ± 12.0	90.8 ± 12.0	90.2 ± 12.4	<0.001
WHR	0.92 ± 0.07	0.91 ± 0.07	0.90 ± 0.07	0.90 ± 0.07	<0.001
Systolic BP (mm Hg)	114 ± 16.2	112 ± 15.2	111± 15.2	110 ± 15.4	<0.001
Diastolic BP (mm Hg)	76.7 ± 10.8	75.2 ± 10.3	74.6 ± 10.4	74.7 ± 10.7	<0.001
Total cholesterol (mg/dl)	186 ± 38.8	184 ± 37.5	184 ± 37.1	183 ± 38.2	0.20
TG/HDL-C ratio	3.47 ± 3.08	3.16 ± 2.40	3.18 ± 2.70	2.84 ± 2.14	<0.001
FBG (mg/dl)	93.3 ± 8.84	92.6 ± 8.57	92.7 ± 8.45	91.7 ± 8.33	<0.001

Family history of diabetes n(%Yes)	124 (9.3%)	109 (8.3%)	135 (10.1%)	151 (11.2%)	0.31
Education					
Elementary	200 (15%)	139 (10.6%)	154 (11.5%)	151 (11.2%)	<0.001
Diploma	759 (57%)	764 (58.1%)	809 (60.5%)	849 (60.1%)	
Academic	372 (27.9%)	411 (31.3%)	375 (28%)	345 (25.7%)	

Values are mean ± SD unless otherwise listed. P values were derived from analysis of variance and Chi square test was used for continuous and dichotomous variables, respectively.

BMI: body mass index, MET: Metabolic equivalent, WC: waist circumference, WHR: waist to height ratio, BP: blood pressure, TG: triglyceride, HDL-C: high density lipoprotein ratio, FBG: fasting blood glucose

[a]: An individual who has given up cigarette and/or tobacco smoking.

Table 2. Dietary intake of adult participants across quartiles of spice intake: the Tehran Lipid and Glucose Study.

Variables	Q1	Q2	Q3	Q4	P-value
Carbohydrate (% of energy)	60.2 ± 5.96	59.3 ± 5.33	59.1 ± 5.08	57.9 ± 5.47	<0.001
Protein (% of energy)	15.1 ± 3.68	15.0 ± 2.49	14.9 ± 2.49	14.7 ± 2.81	0.002
Total fat (% of energy)	28.3 ± 7.80	29.1 ± 4.73	29.4 ± 4.65	31.0 ±5.18	<0.001
SFA (% of energy)	10.2 ± 24.5	9.73 ± 2.70	9.82 ± 2.89	10.1 ± 2.76	0.005
PUFA (% of energy)	5.80 ± 6.29	5.86 ± 1.53	6.02± 1.45	6.28 ± 1.66	<0.001
MUFA (% of energy)	10.1 ± 24.5	9.74 ± 2.37	10.1 ± 3.08	10.5 ± 2.64	<0.001
Fiber (gr/1000 kcal)	9.99 ± 2.92	10.0 ± 2.75	10.5 ± 2.82	10.5 ± 3.33	<0.001
Energy intake, (kcal/day)	2172 ± 652	2330 ± 694	2416 ± 711	2494 ± 637	<0.001

Data are Mean ± SD. ANOVA test; P for trends across quartiles of spice consumption were performed by assigning continuous variables in a liner regression model, and chi-square test was applied for categorical variables.

SFA: saturated fatty acid, MUFA: Mono-unsaturated fatty acid, PUFA: poly unsaturated fatty acid

Table 3. HRs (95% CI) of T2D, MetS and its components incidence across categories of spice intake in adult participants of the Tehran Lipid and Glucose Study.

Quartiles of Spice intake						
**Variables**		**Q1**	**Q2**	**Q3**	**Q4**	**P-trend**
**Incidence of MetS (n=4353)**		328	316	288	279	
**Median** (gr/day)		0.26	1.0	2.0	3.0	
HR (95% CI)	[a] Crude	Ref.	0.98 (0.90-1.07)	0.94 (0.87-1.03)	1.08 (0.99-1.18)	0.63
	[b] Model adjusted	Ref.	1.01 (0.91-1.12)	1.02 (0.92-1.14)	1.05 (0.94-1.18)	0.65
**Incidence of Low HDL-C (n=2688)**		267	218	237	245	
**Median ((**gr/day)		0.21	1.02	1.92	3.0	
HR (95% CI)	[a] Crude	Ref.	0.88 (0.79-0.98)	0.88 (0.78-0.98)	0.97 (0.87-1.09)	0.23
	[3]Model adjusted	Ref.	0.95 (0.83-1.07)	0.94 (0.81-1.07)	0.97 (0.83-1.12)	0.91
**Incidence of High FBG (n=4949)**		348	346	312	301	
**Median** (gr/day)		0.26	1.03	1.99	3.0	
HR (95% CI)	[a]Crude	Ref.	0.97 (0.90-1.05)	0.95 (0.88-1.03)	1.08 (1.00-1.17)	0.20
	[3]Model adjusted	Ref.	0.94 (0.85-1.04)	0.95 (0.86-1.05)	1.11 (0.99-1.24)	0.08
**Incidence of high TG**		336	330	317	323	

**(n=4011)**						
**Median (Spice)** (gr/day)		0.26	1.07	2.0	3.0	
HR (95% CI)	[a] Crude	Ref.	1.01 (0.93-1.11)	0.96 (0.88-1.05)	1.10 (1.01-1.21)	0.39
	[3]Model adjusted	Ref.	1.05 (0.94-1.17)	1.09 (0.97-1.23)	1.19 (1.05-1.35)	0.95
**Incidence of High WC (n=3709)**		248	259	237	237	
**Median** (gr/day)		0.27	1.12	2.01	3.00	
HR (95% CI)	[a] Crude	Ref.	0.96 (0.88-1.06)	0.91 (0.83-1.00)	1.00 (0.91-1.10)	0.83
	[c] Model adjusted	Ref.	0.96 (0.85-1.07)	0.93 (0.83-1.04)	0.98 (0.86-1.11)	0.98
**Incidence High BP (n=4754)**		335	312	330	318	
**Median** (gr/day)		0.26	1.07	2.00	3.00	
HR (95% CI)	[a] Crude	Ref.	0.98 (0.90-1.06)	1.00 (0.92-1.08)	1.16 (1.07-1.26)	0.002
	[c] Model adjusted	Ref.	1.03 (0.93-1.14)	1.06 (0.96-1.17)	1.16 (1.04-1.30)	0.007
**Incidence T2D (n)**		101	99	98	109	
**Median** (gr/day)		0.26	0.96	1.8	3.0	
HR (95% CI)	[a] Crude	Ref.	0.99 (0.92-1.0)	1.0 (0.94-1.1)	1.1 (1.0-1.1)	0.01
	[d] Model adjusted	Ref.	1.0 (0.91-1.1)	0.99 (0.90-1.0)	1.0 (0.94-1.1)	0.24

The median of each quartile was utilized as a continuous variable to estimate the overall trends of HRs across quartiles of spice in the Cox proportional hazard regression models.

[a]: Crude Model adjusted for age, sex

[b]: Model adjusted for age, sex, BMI, smoking, education level, physical activity, total energy, saturated fat (percentage of energy), and fiber intake (gr/1000 kcal).

[c]: In models to estimate the HR of high BP, high TG, high FBG, and low HDL-C respectively, the continuous amount of each risk factor at the beginning of study corresponding to each model was added to the adjustment factors.

[d]: Model adjusted for age, sex, diabetes risk score, physical activity, smoking, education level, total energy intake, saturated fat (percentage of energy), and fiber intake (gr/1000 kcal).

FBS: fasting blood sugar, HDL-C: high density lipoprotein, TG: triglyceride, WC: waist circumference, BP: blood pressure, T2D: type 2 diabetes

Table 4. HRs (95% CI) of T2D, MetS and its components incidence across categories of pepper intake in adult participants of the Tehran Lipid and Glucose Study.

Quartiles of Pepper*						
Variables		Q1	Q2	Q3	Q4	P-trend
**Incidence of MetS (n=4353)**		260	324	329	298	
**Median** (gr/day)		0.88	3.2	8.2	19.7	
HR (95% CI)	[a] Crude	Ref.	1.06 (0.97-1.15)	0.99 (0.91-1.08)	1.07 (0.98-1.17)	0.34
	[b] Model adjusted	Ref.	0.98 (0.88-1.09)	1.00 (0.90-1.11)	1.02 (0.91-1.13)	0.51
**Incidence of Low HDL-C (n=2688)**		234	237	236	260	
**Median** (gr/day)		0.90	3.19	8.23	19.9	
HR (95% CI)	[a] Crude	Ref	0.97 (0.87-1.09)	0.87 (0.78-0.97)	1.03 (0.92-1.14)	0.008
	[c] Model adjusted	Ref	0.91 (0.79-1.05)	0.89 (0.78-1.01)	1.01 (0.88-1.16)	0.24
**Incidence of High FBS (n=4949)**		305	355	342	305	
**Median** (gr/day)		0.90	3.19	8.33	19.7	
HR (95% CI)	[a] Crude	Ref	1.01 (0.93-1.09)	0.93 (0.86-1.01)	0.98 (0.90-1.06)	0.88
	[c] Model adjusted	Ref	0.98 (0.88-1.08)	0.94 (0.85-1.04)	0.96 (0.87-1.07)	0.54
**Incidence of High TG**		300	326	343	337	

**(n=4011)**						
**Median** (gr/day)		0.81	3.19	8.32	19.8	
HR (95% CI)	[a] Crude	Ref	1.00 (0.92-1.09)	0.98 (0.90-1.07)	1.03 (0.94-1.13)	0.50
	[c] Model adjusted	Ref	0.94 (0.84-1.05)	0.99 (0.88-1.10)	0.99 (0.88-1.11)	0.31
**Incidence of High WC (n=3709)**		231	243	247	260	
**Median**		0.89	3.19	8.21	19.7	
HR (95% CI)	[1]Crude	Ref	0.99 (0.91-1.09)	0.95 (0.87-1.04)	1.04 (0.95-1.14)	0.29
	[c] Model adjusted	Ref	0.92 (0.82-1.04)	0.92 (0.82-1.03)	0.94 (0.83-1.06)	0.21
**Incidence High BP (n=4754)**		291	351	352	301	
**Median** (gr/day)		0.89	3.19	8.22	19.7	
HR (95% CI)	[a] Crude	Ref	1.04 (0.96-1.13)	1.00 (0.92-1.09)	1.02 (0.94-1.11)	0.57
	[c] Model adjusted	Ref	0.91 (0.82-1.01)	0.97 (0.88-1.07)	0.89 (0.80-0.99)	0.20
**Incidence T2D (n)**		107	95	103	101	
**Median** (gr/day)		1.4	4.0	8.6	19.7	
HR (95% CI)	[a] Crude	Ref	0.97 (0.90-1.0)	0.96 (0.89-1.0)	0.95(0.88-1.0)	0.80
	[d] Model adjusted	Ref	0.98 (0.89-1.0)	1.0 (0.91-1.0)	0.98 (0.89-1.0)	0.86

The median of each quartile was utilized as a continuous variable to estimate the overall trends of HRs across quartiles of pepper intake in the Cox proportional hazard regression models

[a]: Crude Model adjusted for age, sex

[b]: Model adjusted for age, sex, BMI, smoking, education level, physical activity, total energy, saturated fat (percentage of energy), and fiber intake (gr/1000 kcal).

[c]: In models to estimate the HR of high BP, high TG, high FBG, and low HDL-C respectively, the continuous amount of each risk factor at the beginning of study corresponding to each model was added to the adjustment factors.

[d]: Model adjusted for age, sex, diabetes risk score, physical activity, smoking, education level, total energy intake, saturated fat (percentage of energy), and fiber intake (gr/1000 kcal).

FBS: fasting blood sugar, HDL-C: high density lipoprotein, TG: triglyceride, WC: waist circumference, BP: blood pressure, T2D: type 2 diabetes

*Pepper = Bell pepper + green pepper

Discussion:

This study was conducted to evaluate the association of spice and pepper intakes with T2D, MetS and its components incidence. Our result showed that spice and pepper intakes didn't associate with T2D and MetS. Although, spice intake significantly increased T2D incidence in the crude model. Also, by increasing spice intake, the hazard ratio of high BP and high TG increased. In addition, pepper intake significantly improved low HDL-C in the crude model.

Until now, no prospective study has been performed to investigate the relationship of spice and pepper intakes with the incidence of diabetes and MetS.

An RCT was conducted to evaluate the effect of Turmeric (2.4 g/day) and Black seed (1.5 g/day) in combination (900 mg Black seeds and 1.5 g Turmeric/day) or alone for 8 weeks in patients with MetS. The results showed that combination therapy can improve CRP, LDL-C, HDL-C, FBG, and total cholesterol in comparison to the placebo group. As well, Turmeric significantly decreased CRP, LDL-C, and total cholesterol. Black seed reduced TG and total cholesterol, significantly [32]. Our data revealed that pepper or spice consumption did not associate with MetS incidence; however, our findings showed that pepper intake can improve the hazard ratio of low HDL-C levels. Also, our results showed that in the upper quartile of spice intake the hazard ratio of high TG level increased, remarkably. As well, the findings showed that by increasing spice intake, fat consumption has been increased. Although, we have adjusted to control the intake of fat, it is possible that have not been able to control the effects of excessive fat consumption, resulting in an increase in triglycerides. These findings suggest that the specific amount and type of spice or pepper can result in different effects.

Oxidative stress and inflammatory conditions are related to the MetS. Cinnamon (*Cinnamomum*) regulates gene expression of pro-inflammatory and anti-inflammatory and glucose transporters in

mouse macrophages and adipocytes. Also, curcumin derived from turmeric (*Curcuma longa*), increased glucose uptake by insulin sensitivity improvement in 3T3-L1 cells. Further, curcumin has beneficial anti-inflammatory effects and glucose regulatory properties by suppressing Tumor necrosis factor (TNF) and IL-6 transcription and secretion [33].

A prospective cohort study showed that frequency and average amount of spicy food consumption were inversely associated with LDL-C to cholesterol ratio. Also, this study suggests that spicy food may be another choice to diminish the risk of CVD. It suggests that bioactive components in spicy food can justify these metabolic effects. However, frequency and the average amount of spicy foods have a positive relation with triacylglycerol (TAG) [34]. In contrast, another clinical study demonstrated that the chili diet has no remarkable effects on lipid metabolism after 3 weeks of intervention [35]. Glucose uptake is facilitated by AMPK or PI3K or MAPK signaling pathways. Also, curcumin activates peroxisome proliferator-activated receptor (PPAR) leading to suppression of LDL-C receptor gene expression [36].

A meta-analysis showed that ginger in various types (tablet, capsules, powder, or rhizomes) significantly decreased FBG and TG, and increased HDL-C levels [37]. Another meta-analysis showed that cinnamon significantly decreased LDL-C, cholesterol, TG, and elevated HDL-C levels, although HbA1C had no remarkable change [38]. Cellular cholesterol reduction decreases fatty acid synthesis by inhibiting fatty acid synthase. Cholesterol reduction defeats adipocyte differentiation, and lipid accumulation which attributed to curcumin benefits (34). Herbs and spices may have beneficial metabolic effects on AMPK activating, including fatty acid oxidation and mitochondrial functions that contribute to attenuating MetS symptoms (35).

In a similar study, the intake of polyphenols and flavonoids from fruits and vegetables with the incidence of gestational diabetes mellitus (GDM) has been evaluated. The results showed that

total dietary intake of polyphenols and flavonoids from fruits decreased the risk of GDM, while the polyphenols and flavonoids in vegetables didn't relate to GDM. This reduction is mainly associated with 2-h FBG instead of FBG and 1-h FBG [39]. However, our finding didn't show any relation between spice consumption and diabetes incidence. Spices have polyphenols that can affect the metabolism of glucose, including intestinal glucose absorption, insulin secretion from B-cells in the pancreas, regulation of glucose liberation from the liver, increased expression of insulin receptors, and glucose uptake from tissues that are sensitive to insulin [40]. However, these effects can alter during the process of digestion and absorption. Furthermore, the most polyphenols contained in spices has no sufficient absorption, and plasma levels are transient due to the rapid metabolism of these compounds. The complexity effects of spices on diabetes has been determined by the fact that different spices contain different phenolic compounds and synergistic effects [41].

LV et al. study [42] reported that the consumption of spicy food strongly reverses certain causes of mortality independent of other risk factors of death in women and men. Also, this study illustrated that those who consumed spicy food 6 or 7 days a week, had a 14% reduction in total mortality rate; however, this association was stronger in individuals who didn't consume alcohol rather than those who consumed it.

As a result, the effects of spices on diabetes can vary significantly based on the types and combinations of spice used. Hence, it is crucial to consider the specific types and mixtures of spice when evaluating their impact on diabetes [43]. Thus, it can be inferred that the type of spice utilized, the timing of its addition to the food, and the cooking methods employed can influence the polyphenols and their antioxidant properties. These factors may lead to diverse outcomes in research studies.

Similar to a previous study, a prospective study was conducted to investigate the association between total polyphenol intake and its subclasses with diabetes incidence. Results indicated that the intake of polyphenols, phenolic acids, and lignins had an inverse relation with T2D. This investigation also showed that several polyphenols can decrease the incidence of diabetes [44]. As a result of two previous studies, such polyphenols (not all of them) can reduce diabetes incidence.

Hashemian et al. [45] conducted a prospective cohort study to evaluate the association of some spice consumption (turmeric, black or chili pepper, cinnamon, and saffron) with all and certain cause mortality in Iran. The findings showed that turmeric and saffron consumption were related to a significant reduction in overall mortality and cardiovascular mortality. Furthermore, black or chili pepper was associated with a significant reduction in risk of overall mortality. Also, no spices have shown an association with the cancer mortality. In our study, we evaluated the spices that people commonly consumed. However, in Hashemian's research, the influence of specific spices on certain aspects of mortality rates was assessed. Also, our results showed that BP significantly increase in upper quartiles of spices. As well, the individuals who consumed more spices tended to have a higher fat intake. Based on our results, this can justify that other components of foods can change the benefit of spices and may change polyphenol and antioxidant effects leading to undesirable results. A meta-analysis represented that the intake of a remarkable amount of phenolic acid (600 mg per day) is related to a 34% reduction in the risk of T2D. Thus, we conclude that the dosage of polyphenol consumption is important for its effectiveness [46]. Perhaps, in our study, the inadequate amount of spice intake leading to unremarkable results.

In an RCT, the consumption of cinnamon with a dosage of 1, 3, and 6 grams per day can significantly decrease serum levels of Glucose, TG, and LDL-C compared to placebo in people with T2D [47]. Other RCT assessed the effect of ginger consumption (3 grams/day) on glycemic indices. The result of this study indicated that ginger can reduce FBS and HBA1C and elevate the QUICKI index in the treatment group compared to the placebo group [48].

Also, Chuengsamarn et al. [49] conducted an RCT to evaluate the effect of curcumin supplementation on pre-diabetic patients to prevent diabetic conditions. In this 9-month intervention, the curcumin group exhibits better function of pancreatic B-cells. Also, C-peptide and HOMA-IR were lower in the treatment group, and adiponectin and HOMA-B were higher in the curcumin group. As a result, the curcumin intervention lowered the development of pre-diabetic to diabetes conditions.

In an in vitro study, the effect of some high spice consumption on hemoglobin glycation has been assessed. The results suggest that wild caraway, turmeric, cardamom and, black pepper may prevent hemoglobin glycation. However, some extracts such as anise and saffron have both inhibitory and pro-glycation properties, as well. Based on the results obtained, wild caraway, turmeric, cardamom, and black pepper particularly wild caraway extracts are potent antiglycation agents, which can contribute to the prevention of diabetic complications associated with glycation [50]. As a consequence, results showed that various factors can affect on bioavailability and absorption of the polyphenol or antioxidant activity of spices. So, further studies are needed to approve these matters.

Our result exhibited several notable strengths. First, the population-based Tehran Lipid and Glucose Study (TLGS) provided robust data on the urban population of Tehran. Second, the diagnosis of diabetes was based on a fasting blood experiment, ensuring greater reliability. Third,

the calculation for the nutrient content of food items was conducted cumulatively across all follow-up surveys beginning from the baseline survey up to the last follow-up visit where diabetes was diagnosed. Lastly, we thoroughly evaluated all aspects of dietary factors, including fiber and fatty acids, in relation to the onset of diabetes and accounted for any possible confounding factors in our final analysis.

We must acknowledge several limitations in our study. Firstly, we lacked data on the common methods of using spices and the time of adding them to food, and thus, failed to analyze their impact on the development of T2D and Mets. Secondly, despite our efforts to identify all cofounding may not have been eliminated due to knowledge or measurement gaps. Furthermore, since insulin sensitivity- a highly sensitive marker- was not available for all participants, we were unable to detect the association between spice and pepper consumption and the risk of insulin resistance. Dietary information was collected by expert dietitians without the use of food replicas, and previous studies that utilized the food frequency questionnaire (FFQ) reported significant under-reporting of energy intake at group levels, ranging from 4.6 to 42%, in comparison to the doubly labeled water method [51]. The degree of misreporting in our research fell within this range.

Conclusion:

Finally, our findings showed that spice and pepper intakes had no association with the incidence of T2D and Mets. In addition, the hazard ratio of low HDL-C improved in the third quartile of pepper. Also, by increasing spice intake the hazard ratio of BP and TG levels significantly increased.

Acknowledgments:

The authors would like to thank the participants and TLGS personal for their collaboration. Authors' contributions: AI and FHE collected the data and wrote the first draft, statistical analysis: AI and SHD, critical revision of the manuscript: FH-E, PM and FA. All the authors have given final approval of the version to be published.

Conflict of interest:

The authors declare that they have no competing interests.

Funding:

This work was supported by the Research Institute for Endocrine Science, Shahid Beheshti University of Medical Science (Tehran, Iran) under Grant number. 43003670.

References:

1. Mohamed SM, Shalaby MA, El-Shiekh RA, El-Banna HA, Emam SR, Bakr AF. Metabolic syndrome: risk factors, diagnosis, pathogenesis, and management with natural approaches. Food Chemistry Advances. 2023;3:100335.
2. Fahed G, Aoun L, Bou Zerdan M, Allam S, Bou Zerdan M, Bouferraa Y, Assi HI. Metabolic syndrome: updates on pathophysiology and management in 2021. International journal of molecular sciences. 2022;23(2):786.
3. Shi Y, Hu FB. The global implications of diabetes and cancer. Lancet (London, England). 2014;383(9933):1947-8.
4. Li Z, Geng Y-N, Jiang J-D, Kong W-J. Antioxidant and anti-inflammatory activities of berberine in the treatment of diabetes mellitus. Evidence-based complementary and alternative medicine. 2014;2014.
5. Barouti AA, Tynelius P, Lager A, Björklund A. Fruit and vegetable intake and risk of prediabetes and type 2 diabetes: results from a 20-year long prospective cohort study in Swedish men and women. Eur J Nutr. 2022;61(6):3175-87.
6. Pereira AS, Banegas-Luna AJ, Peña-García J, Pérez-Sánchez H, Apostolides Z. Evaluation of the anti-diabetic activity of some common herbs and spices: Providing new insights with inverse virtual screening. Molecules. 2019;24(22):4030.
7. S Panickar K. Beneficial effects of herbs, spices and medicinal plants on the metabolic syndrome, brain and cognitive function. Central Nervous System Agents in Medicinal Chemistry (Formerly Current Medicinal Chemistry-Central Nervous System Agents). 2013;13(1):13-29.
8. Matsui T, Ueda T, Oki T, Sugita K, Terahara N, Matsumoto K. alpha-Glucosidase inhibitory action of natural acylated anthocyanins. 1. Survey of natural pigments with potent inhibitory activity. J Agric Food Chem. 2001;49(4):1948-51.
9. Vázquez-Ruiz Z, Martínez-González M, Vitelli-Storelli F, Bes-Rastrollo M, Basterra-Gortari FJ, Toledo E. Effect of Dietary Phenolic Compounds on Incidence of Type 2 Diabetes in the "Seguimiento Universidad de Navarra" (SUN) Cohort. Antioxidants (Basel). 2023;12(2).
10. Chen L, Pu Y, Xu Y, He X, Cao J, Ma Y, Jiang W. Anti-diabetic and anti-obesity: Efficacy evaluation and exploitation of polyphenols in fruits and vegetables. Food Res Int. 2022;157:111202.
11. Cazzola R, Camerotto C, Cestaro B. Anti-oxidant, anti-glycant, and inhibitory activity against α-amylase and α-glucosidase of selected spices and culinary herbs. International Journal of food sciences and nutrition. 2011;62(2):175-84.
12. Suhaj M. Spice antioxidants isolation and their antiradical activity: a review. Journal of food composition and analysis. 2006;19(6-7):531-7.
13. Yi W, Wetzstein HY. Biochemical, biological and histological evaluation of some culinary and medicinal herbs grown under greenhouse and field conditions. Journal of the Science of Food and Agriculture. 2010;90(6):1063-70.
14. Shan B, Cai YZ, Sun M, Corke H. Antioxidant capacity of 26 spice extracts and characterization of their phenolic constituents. Journal of agricultural and food chemistry. 2005;53(20):7749-59.
15. Elosta A, Ghous T, Ahmed N. Natural products as anti-glycation agents: possible therapeutic potential for diabetic complications. Current diabetes reviews. 2012;8(2):92-108.
16. Liu Y-J, Zhan J, Liu X-L, Wang Y, Ji J, He Q-Q. Dietary flavonoids intake and risk of type 2 diabetes: a meta-analysis of prospective cohort studies. Clinical Nutrition. 2014;33(1):59-63.

17. Cao H, Ou J, Chen L, Zhang Y, Szkudelski T, Delmas D, et al. Dietary polyphenols and type 2 diabetes: Human Study and Clinical Trial. Critical reviews in food science and nutrition. 2019;59(20):3371-9.
18. Alkhatib DH, Jaleel A, Tariq MNM, Feehan J, Apostolopoulos V, Cheikh Ismail L, et al. The Role of Bioactive Compounds from Dietary Spices in the Management of Metabolic Syndrome: An Overview. Nutrients. 2021;14(1).
19. Willett W. Issues in analysis and presentation of dietary data. Nutritional epidemiology. 1998:321-46.
20. AZIZI F, Hadaegh F, KHALILI D, Esteghamati A, HOSSEIN PF, Delavari A, et al. Appropriate definition of metabolic syndrome among Iranian adults: report of the Iranian National Committee of Obesity. 2010.
21. Azizi F, Ghanbarian A, Momenan AA, Hadaegh F, Mirmiran P, Hedayati M, et al. Prevention of non-communicable disease in a population in nutrition transition: Tehran Lipid and Glucose Study phase II. Trials. 2009;10:5.
22. Azizi F, Rahmani M, Emami H, Mirmiran P, Hajipour R, Madjid M, et al. Cardiovascular risk factors in an Iranian urban population: Tehran lipid and glucose study (phase 1). Soz Praventivmed. 2002;47(6):408-26.
23. Esfahani FH, Asghari G, Mirmiran P, Azizi F. Reproducibility and relative validity of food group intake in a food frequency questionnaire developed for the Tehran Lipid and Glucose Study. J Epidemiol. 2010;20(2):150-8.
24. Mirmiran P, Esfahani FH, Mehrabi Y, Hedayati M, Azizi F. Reliability and relative validity of an FFQ for nutrients in the Tehran lipid and glucose study. Public Health Nutr. 2010;13(5):654-62.
25. Farhadnejad H, Teymoori F, Asghari G, Mokhtari E, Mirmiran P, Azizi F. The higher adherence to a healthy lifestyle score is associated with a decreased risk of type 2 diabetes in Iranian adults. BMC Endocrine Disorders. 2022;22(1):42.
26. Momenan AA, Delshad M, Sarbazi N, Rezaei Ghaleh N, Ghanbarian A, Azizi F. Reliability and validity of the Modifiable Activity Questionnaire (MAQ) in an Iranian urban adult population. Arch Iran Med. 2012;15(5):279-82.
27. Ainsworth BE, Haskell WL, Whitt MC, Irwin ML, Swartz AM, Strath SJ, et al. Compendium of physical activities: an update of activity codes and MET intensities. Med Sci Sports Exerc. 2000;32(9 Suppl):S498-504.
28. Azizi F, Ghanbarian A, Momenan AA, Hadaegh F, Mirmiran P, Hedayati M, et al. Prevention of non-communicable disease in a population in nutrition transition: Tehran Lipid and Glucose Study phase II. Trials. 2009;10(1):1-15.
29. Bozorgmanesh M, Hadaegh F, Ghaffari S, Harati H, Azizi F. A simple risk score effectively predicted type 2 diabetes in Iranian adult population: population-based cohort study. Eur J Public Health. 2011;21(5):554-9.
30. 2. Classification and Diagnosis of Diabetes: Standards of Medical Care in Diabetes-2019. Diabetes Care. 2019;42(Suppl 1):S13-s28.
31. Grundy SM, Hansen B, Smith Jr SC, Cleeman JI, Kahn RA, Participants C. Clinical management of metabolic syndrome: report of the American Heart Association/National Heart, Lung, and Blood Institute/American Diabetes Association conference on scientific issues related to management. Circulation. 2004;109(4):551-6.
32. Amin F, Islam N, Anila N, Gilani AH. Clinical efficacy of the co-administration of Turmeric and Black seeds (Kalongi) in metabolic syndrome - a double blind randomized controlled trial - TAK-MetS trial. Complement Ther Med. 2015;23(2):165-74.

33. Wang SL, Li Y, Wen Y, Chen YF, Na LX, Li ST, Sun CH. Curcumin, a potential inhibitor of up-regulation of TNF-alpha and IL-6 induced by palmitate in 3T3-L1 adipocytes through NF-kappaB and JNK pathway. Biomed Environ Sci. 2009;22(1):32-9.
34. Xue Y, He T, Yu K, Zhao A, Zheng W, Zhang Y, Zhu B. Association between spicy food consumption and lipid profiles in adults: a nationwide population-based study. Br J Nutr. 2017;118(2):144-53.
35. Ahuja KD, Robertson IK, Geraghty DP, Ball MJ. The effect of 4-week chilli supplementation on metabolic and arterial function in humans. Eur J Clin Nutr. 2007;61(3):326-33.
36. Kang C, Kim E. Synergistic effect of curcumin and insulin on muscle cell glucose metabolism. Food Chem Toxicol. 2010;48(8-9):2366-73.
37. Jafarnejad S, Keshavarz SA, Mahbubi S, Saremi S, Arab A, Abbasi S, Djafarian K. Effect of ginger (Zingiber officinale) on blood glucose and lipid concentrations in diabetic and hyperlipidemic subjects: A meta-analysis of randomized controlled trials. Journal of functional foods. 2017;29:127-34.
38. Allen RW, Schwartzman E, Baker WL, Coleman CI, Phung OJ. Cinnamon use in type 2 diabetes: an updated systematic review and meta-analysis. Ann Fam Med. 2013;11(5):452-9.
39. Gao Q, Zhong C, Zhou X, Chen R, Xiong T, Hong M, et al. Inverse association of total polyphenols and flavonoids intake and the intake from fruits with the risk of gestational diabetes mellitus: A prospective cohort study. Clin Nutr. 2021;40(2):550-9.
40. Bi X, Lim J, Henry CJ. Spices in the management of diabetes mellitus. Food Chem. 2017;217:281-93.
41. Cazzola R, Cestaro B. Antioxidant spices and herbs used in diabetes. Diabetes: Oxidative stress and dietary antioxidants: Elsevier; 2014. p. 89-97.
42. Lv J, Qi L, Yu C, Yang L, Guo Y, Chen Y, et al. Consumption of spicy foods and total and cause specific mortality: population based cohort study. Bmj. 2015;351.
43. Sanlier N, Gencer F. Role of spices in the treatment of diabetes mellitus: A minireview. Trends in food science & technology. 2020;99:441-9.
44. Esfandiar Z, Hosseini-Esfahani F, Mirmiran P, Yuzbashian E, Azizi F. The Association of Dietary Polyphenol Intake with the Risk of Type 2 Diabetes: Tehran Lipid and Glucose Study. Diabetes Metab Syndr Obes. 2020;13:1643-52.
45. Hashemian M, Murphy G, Etemadi A, Abnet C, Malekzadeh R. Turmeric, Pepper, Cinnamon, and Saffron Consumption and Mortality (P18-069-19). Curr Dev Nutr. 2019;3(Suppl 1).
46. Rienks J, Barbaresko J, Oluwagbemigun K, Schmid M, Nöthlings U. Polyphenol exposure and risk of type 2 diabetes: dose-response meta-analyses and systematic review of prospective cohort studies. Am J Clin Nutr. 2018;108(1):49-61.
47. Khan A, Safdar M, Ali Khan MM, Khattak KN, Anderson RA. Cinnamon improves glucose and lipids of people with type 2 diabetes. Diabetes Care. 2003;26(12):3215-8.
48. Shidfar F, Rajab A, Rahideh T, Khandouzi N, Hosseini S, Shidfar S. The effect of ginger (Zingiber officinale) on glycemic markers in patients with type 2 diabetes. J Complement Integr Med. 2015;12(2):165-70.
49. Chuengsamarn S, Rattanamongkolgul S, Luechapudiporn R, Phisalaphong C, Jirawatnotai S. Curcumin extract for prevention of type 2 diabetes. Diabetes Care. 2012;35(11):2121-7.
50. Naderi GH, Dinani NJ, Asgary S, Taher M, Nikkhoo N, Boshtam M. Effect of some high consumption spices on hemoglobin glycation. Indian J Pharm Sci. 2014;76(6):553-7.
51. Burrows TL, Ho YY, Rollo ME, Collins CE. Validity of Dietary Assessment Methods When Compared to the Method of Doubly Labeled Water: A Systematic Review in Adults. Front Endocrinol (Lausanne). 2019;10:850.

Supporting information:

Supplementary Table 1 (S1)**:** Baseline characteristics of adult participants across quartiles of spice intake: the Tehran Lipid and Glucose Study (Mets).

Table S2**.** Dietary intake in the quartiles of spices intake (Mets).

Figure Click here to access/download;Figure;fig1.tiff

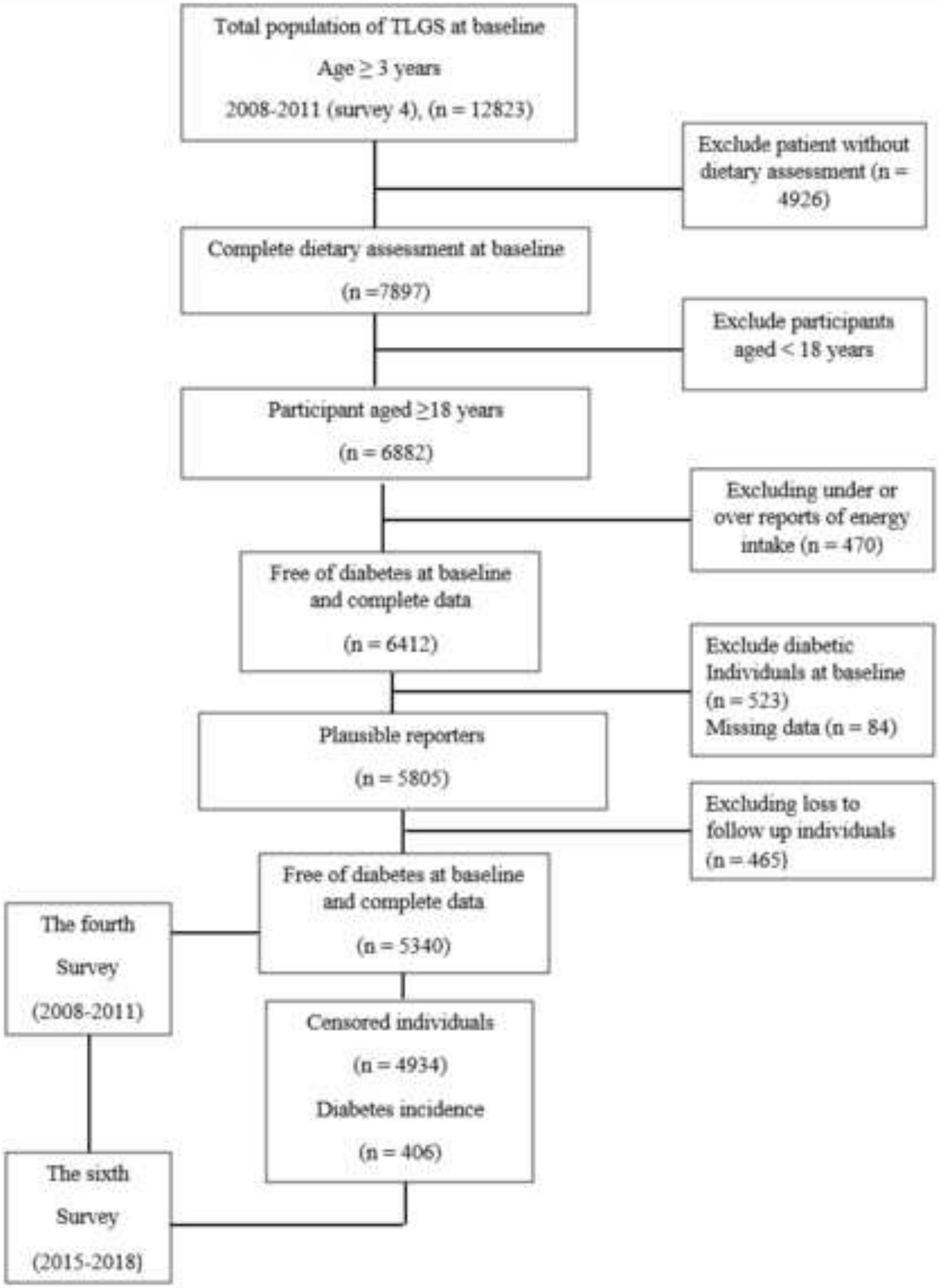

Figure 1.

Click here to access/download;Figure;fig 2.tiff

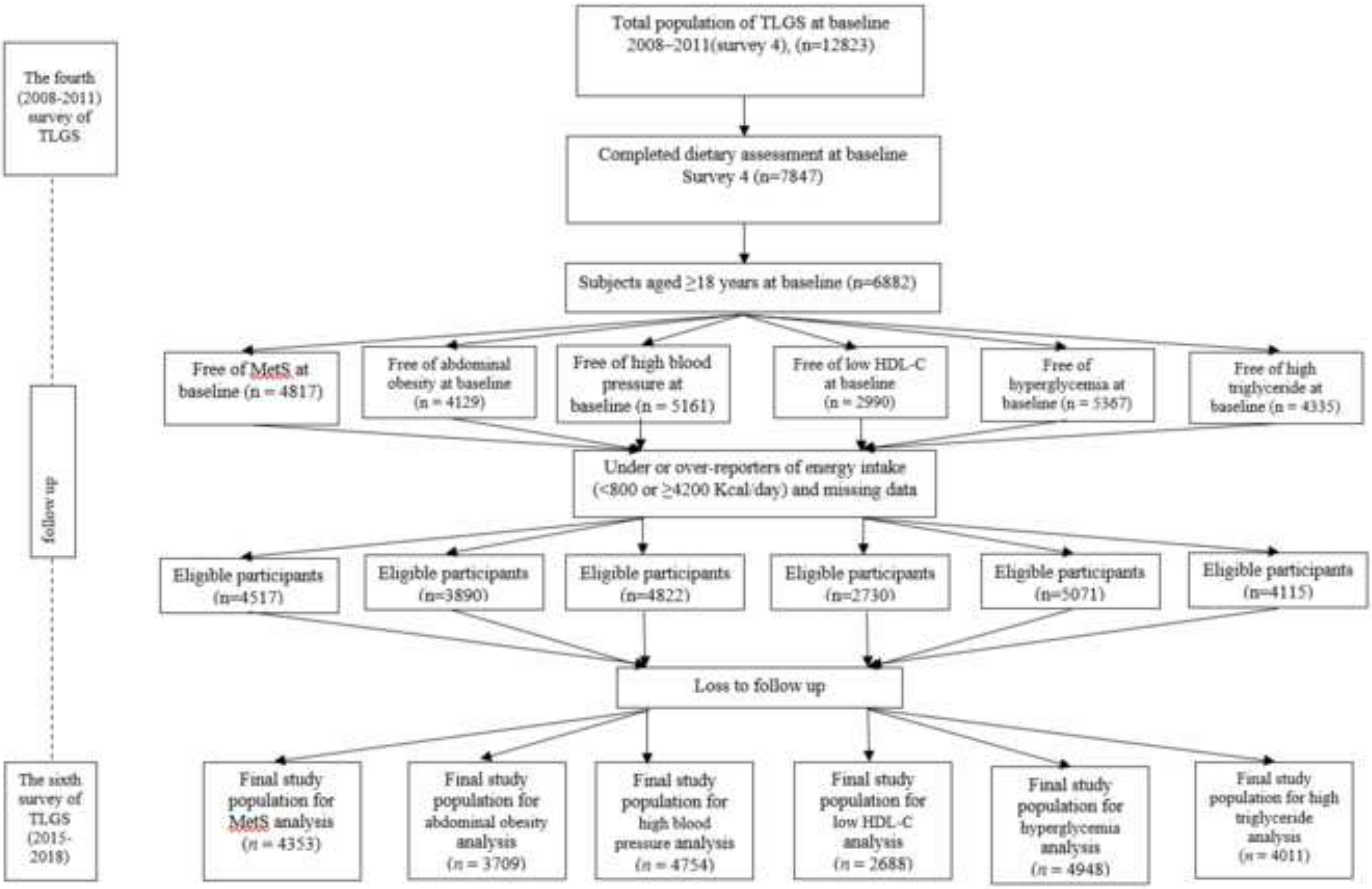

Figure 2.

Click here to access/download
Supporting Information
supplementary file.docx
