# Supplementary material for: Associations between spice or pepper (Capsicum annuum) consumption and diabetes or metabolic syndrome incidence
Source: PLoS One. 2025 Feb 11;20(2):e0314448. doi: 10.1371/journal.pone.0314448 (PMC11813124; doi:10.1371/journal.pone.0314448)
Supplement: Attachment — Submitted filename: Review PONE-D-24-24401.docx [file pone.0314448.s004.docx]

Review PONE-D-24-24401

Line 50: Suggest to rather use …and many more than and etc.

Line 51: How does MetS manifests with WC – is there a certain reference value? Or will abdominal obesity be more specific?

Line 61-61: Improve the sentence highlighted in yellow Further, the using of herbs and spices has been paid more attention, while the modern medicine has been improved.

Line 92: Add the word highlighted in red. Of individuals participating in survey 4, 6882 subjects aged ≥18 years were? randomly selected for dietary assessment.

Line 101-102: Add the words highlighted in red. Subjects participating in survey 4 (baseline), aged ≥18 years, and who completed the dietary assessment were entered in this study for MetS secondary analysis.

Line 106: Add the word highlighted in red. …the baseline population and they were tracked until survey 6.

Line 102-104: Subjects with MetS diagnosis at baseline [20], pregnant or lactating women were excluded. Also, individuals with over- or under reporting of energy intake (≥4,200 or <800 kcal/day) were omitted [19] – how is this different form line 93-97? Or is this a repeat?

Line 200-201: Is the fat included there total fat?

Table 1: What are the cut-offs for the different quartiles? What was the average spice intake per quartile? Does the spice intake include the pepper intake as well? Please amend the table title to include peppers. Should you merge the cells for the p-value for education?

Table 2&3 footnote: HR explanation/definition

Table 4: Should it be Quartiles of Pepper intake*

Line 309: replace didn’t with did not

Line 324-326: Add the words highlighted in red. Although, we have adjusted to control for the intake of fat, it is possible that we have not been able to control for the effects of excessive fat consumption, resulting in an increase in triglycerides.

Line 354: replace didn’t with did not

Line 355: replace didn’t with did not

Line 360: …these effects can be altered during the process of digestion and absorption

Line 360: The complexity effects of spices on ~~diabetes~~ T2D has

Line 367: 367 illustrated that those who consumed spicy food ~~6~~ six or ~~7~~ seven days a week

Line 368: replace didn’t with did not

Line 396: Perhaps, in our study, the inadequate amount of spice intake ~~leading~~ led to unremarkable results.

Line 401: HBA1C replace with HbA1c, check the rest of the manuscript for correct format.

Line 429: Add the word highlighted in red. Secondly, despite our efforts to identify all cofounding this may not have been eliminated due to knowledge or measurement gaps.

Line 439: …of T2D and ~~Mets~~ MetS.

FBS vs FBG can you use it interchangeable. The first part of the article it is FBG and at the results it becomes FBS.
