# Supplementary material for: Associations between spice or pepper (Capsicum annuum) consumption and diabetes or metabolic syndrome incidence
Source: PLoS One. 2025 Feb 11;20(2):e0314448. doi: 10.1371/journal.pone.0314448 (PMC11813124; doi:10.1371/journal.pone.0314448)
Supplement: Attachment — Submitted filename: Response ro Reviewers.docx [file pone.0314448.s006.docx]

**Dear Dr. Guoying Wang**

I would like to express my gratitude for your valuable time and thoughtful comments on our manuscript titled “Associations between Spice or Pepper (*Capsicum annuum*) Consumption and Diabetes or Metabolic Syndrome Incidence.” Your insights have been instrumental in refining our work.

In response to your suggestions, we have diligently revised the manuscript. To facilitate your review, we have provided a point-by-point summary of the requested changes, and the modifications are highlighted using the track changes feature in the submitted file.

We are hopeful that the revised manuscript now aligns with the standards for publication in the "PLOS One." Your interest in our research and the opportunity to enhance its quality are greatly appreciated. We remain open to any additional feedback or further revisions that you may recommend ensuring the manuscript meets the journal's criteria.

Once again, thank you for your commitment to advancing scientific discourse, and we look forward to your feedback on the revised version.

Please find the below comments/questions by the editor/reviewers (in black color) and our response/clarification/explanation to the comments and questions (in blue color).

Best regards,

Parvin Mirmiran, Prof.

Nutrition and Endocrine Research Center, Research Institute for Endocrine Sciences, Shahid Beheshti University of Medical Sciences, Tehran, Iran

E-mail: parvin.mirmiran@sbmu.ac.ir

[Parvin.mirmiran@gmail.com](mailto:Parvin.mirmiran@gmail.com)

Journal Requirements:

Please review your reference list to ensure that it is complete and correct. If you have cited papers that have been retracted, please include the rationale for doing so in the manuscript text, or remove these references and replace them with relevant current references. Any changes to the reference list should be mentioned in the rebuttal letter that accompanies your revised manuscript. If you need to cite a retracted article, indicate the article’s retracted status in the References list and also include a citation and full reference for the retraction notice.

Reply: It has been checked.

Reviewer #1:

The authors have addressed most of my concerns. One additional revision is to add to the limitations in the discussion that the method of assessing spice intake was not validated or tested for reliability/repeatability.

Reply|: Based one your request, it has been added to discussion. Also, the method of assessing spice intake was not validated or tested for reliability/repeatability. Page 27, line 446-447.

Reviewer #2:

Line 130-131: Portion sizes were then converted to mass (gr/day) from the household measurements. Would this be a better statement?

Reply: This sentence has been changed based your comment. Page 7, line 130-131.

Line 133: National foods not listed in the USDA FCT were provided from the Iranian FCT. Would this be a better statement?

Reply: Based on your suggestion, this sentence has been changed. Page 7, line 133.

Line 228: This sentence should probably read: Furthermore, increased spice consumption was associated with lower systolic and diastolic BP (P<0.001). The study design is not able to support causality.

Reply: It has been changed. Page 11-12, line 228-229.

Line 360 and 414: HbA1c not corrected.

Reply: It has been corrected. Page 24, line 361.
