# Supplementary material for: Associations between spice or pepper (Capsicum annuum) consumption and diabetes or metabolic syndrome incidence
Source: PLoS One. 2025 Feb 11;20(2):e0314448. doi: 10.1371/journal.pone.0314448 (PMC11813124; doi:10.1371/journal.pone.0314448)
Supplement: Attachment — Submitted filename: FFQ147 items (1).doc [file pone.0314448.s007.doc]

**بسمه تعالي**

**دانشگاه علوم پزشكي و خدمات بهداشتي درماني شهيد بهشتي**

**پژوهشکده علوم غدد درون‌ريز و متابوليسم**

پرسشنامه بسامد خوراک (FFQ) از پروژه پژوهشي " مرحله چهارم کوهورت تغذيه در مطالعه قند و ليپيد تهران"

# نام و نام خانوادگي: شماره خانوار:🗆🗆🗆🗆🗆 کد پذيرش:🗆🗆🗆🗆🗆 سن:🗆🗆

| رديف | مواد غذايي | مقدار | روز | | هفته | ماه | | | | سال | | **ملاحظات** |
| --- | --- | --- | --- | --- | --- | --- | --- | --- | --- | --- | --- | --- |
| 1 | نان لواش | 1 عدد |  | |  |  | | | |  | |  |
| 2 | نان بربري | 1 عدد |  | |  |  | | | |  | |  |
| 3 | نان سنگك | 1 عدد |  | |  |  | | | |  | |  |
| 4 | نان تافتون | 1 عدد |  | |  |  | | | |  | | ماشيني * مشهدي* |
| 5 | نان باگت | 1 عدد كوچك |  | |  |  | | | |  | |  |
| 6 | برنج پخته | 1بشقاب غذاخوري |  | |  |  | | | |  | | معمولي* پر* |
| 7 | ماكاروني پخته | 1 كفگير |  | |  |  | | | |  | | سرصاف* پر* |
| 8 | سيب‌زميني | 1 عدد متوسط |  | |  |  | | | |  | |  |
| 9 | سيب‌زميني سرخ كرده | 1 خلال |  | |  |  | | | |  | |  |
| 10 | ورميشل پخته  (رشته سوپ) | 1 ليوان |  | |  |  | | | |  | |  |
| 11 | رشته آش | 1 ليوان |  | |  |  | | | |  | |  |
| 12 | بيسكويت (با ذكر نوع) | 1عدد |  | |  |  | | | |  | | نوع:............... |
| 13 | كراكر(بيسكويت ترد) | 1عدد |  | |  |  | | | |  | |  |
| 14 | انواع کيکها | 1 عدد/1 برش متوسط |  | |  |  | | | |  | | (شامل کيک يزدي، کيک خانگي، کلوچه، تي تاپ و....) |
| 15 | ذرت و بلال | 1 عدد متوسط |  | |  |  | | | |  | |  |
| 16 | جو يا بلغور پخته | 1استکان |  | |  |  | | | |  | |  |
| 17 | عدس | 1استکان |  | |  |  | | | |  | |  |
| 18 | لوبيا | 1استکان |  | |  |  | | | |  | |  |
| 19 | نخود | 1استکان |  | |  |  | | | |  | |  |
| 20 | باقلا پخته | 1استکان |  | |  |  | | | |  | |  |
| 21 | سويا | 1استکان |  | |  |  | | | |  | |  |
| 22 | ماش | 1استکان |  | |  |  | | | |  | |  |
| 23 | لپه | 1استکان |  | |  | | |  | |  | |  |
| 24 | گوشت گاو يا گوساله | 1 تكه خورشتي |  | |  | | |  | |  | |  |
| 25 | گوشت گوسفند | 1 تكه خورشتي |  | |  | | |  | |  | |  |
| 26 | گوشت چرخ كرده | 1قاشق غذاخوري |  | |  | | |  | |  | |  |
| 27 | مرغ و جوجه | 1 قطعه متوسط |  | |  | | |  | |  | | ران🗆 سينه* بال *  با پوست🗆 بي پوست🗆 |
| 28 | ماهي (بجز تن) با ذكر نوع | 1 قطعه متوسط (1كف دست كامل) |  | |  | | |  | |  | | مقدار: ...........  نوع:.............. |
| **رديف** | **مواد غذايي** | **مقدار** | **روز** | | **هفته** | | | **ماه** | | **سال** | | **ملاحظات** |
| 29 | تن ماهي (كنسرو) | 2/1 قوطي |  | |  | | |  | |  | | آيا روغن آن دور ريخته مي‌شود؟ بله* خير* |
| 30 | دل و جگر و قلوه | 1 سيخ |  | |  | | |  | |  | |  |
| 31 | همبرگر | 1 عدد |  | |  | | |  | |  | |  |
| 32 | کالباس | 1برش |  | |  | | |  | |  | |  |
| 33 | سوسيس | 1 عدد(آلماني* كوكتل* ) |  | |  | | |  | |  | |  |
| **34** | تخم‌مرغ | 1 عدد |  | |  | | |  | |  | |  |
| **35** | سيرابي و شيردان | 1 قطعه |  | |  | | |  | |  | |  |
| **36** | زبان | 1 عدد كامل |  | |  | | |  | |  | |  |
| **37** | مغز | 1 عدد كامل |  | |  | | |  | |  | |  |
| **38** | كله | 1 کف دست |  | |  | | |  | |  | |  |
| **39** | پاچه | 1 عدد |  | |  | | |  | |  | | اگر مقدار و اجزاي خاصي مصرف مي‌شود ذكر شود |
| **40** | پيتزا | 1 عدد |  | |  | | |  | |  | |  |
| **41** | شير كم‌چرب (کمتر از2%) | 1 ليوان |  | |  | | |  | |  | |  |
| **42** | شير پرچرب (بيشتر يا مساوي2%) | 1 ليوان |  | |  | | |  | |  | |  |
| **43** | شير كاكائو | 1 ليوان |  | |  | | |  | |  | |  |
| **44** | ماست چكيده | 1 قاشق غذاخوري |  | |  | | |  | |  | |  |
| **45** | ماست معمولي | 1 كاسه ماست‌خوري |  | |  | | |  | |  | |  |
| **46** | ماست پرچرب | 1 كاسه ماست‌خوري |  | |  | | |  | |  | |  |
| **47** | پنير | 1 قوطي كبريت |  | |  | | |  | |  | |  |
| **48** | پنير خامه‌اي | 1 قوطي كبريت |  | |  | | |  | |  | |  |
| **49** | دوغ | 1 ليوان |  | |  | | |  | |  | |  |
| **50** | خامه و سرشير | 1قاشق غذاخوري |  | |  | | |  | |  | |  |
| **51** | بستني سنتي | نصف ليوان |  | |  | | |  | |  | | چند ماه سال؟ |
| **52** | بستني غير سنتي | 1عدد |  | |  | | |  | |  | | چند ماه سال؟ |
| **53** | كره | 1 قوطي كبريت |  | |  | | |  | |  | |  |
| **54** | مارگارين | 1 قوطي كبريت |  | |  | | |  | |  |  | |
| **55** | كشك | 1قاشق غذاخوري |  | |  | | |  | |  |  | |
| **56** | كاهو خرد شده | 1ليوان |  | |  | | |  | |  |  | |
| **57** | گوجه‌فرنگي | 1 عدد متوسط |  | |  | | |  | |  |  | |
| **58** | خيار | 1 عدد متوسط |  | |  | | |  | |  |  | |
| **59** | سبزي خوردن | 1 پيش‌دستي |  | |  | | |  | |  |  | |
| **60** | سبزي پخته(آش، پلو، سوپ و غيره) | 1ليوان |  | |  | | |  | |  |  | |
| **61** | كدو حلوايي | 1کف دست |  | |  | | |  | |  |  | |
| **رديف** | **مواد غذايي** | **مقدار** | **روز** | | **هفته** | | | **ماه** | | **سال** | **ملاحظات** | |
| **62** | كدو خورشتي | 1 عدد متوسط |  | |  | | |  | |  |  | |
| **63** | بادمجان پخته | 1 عدد متوسط |  | |  | | |  | |  |  | |
| **64** | كرفس پخته | 1استکان |  | |  | | |  | |  |  | |
| **65** | نخود سبز پخته | 1استکان |  | |  | | |  | |  |  | |
| **66** | لوبيا سبز پخته | 1استکان |  | |  | | |  | |  |  | |
| **67** | هويج خام | 1 عدد متوسط |  | |  | | |  | |  |  | |
| **68** | هويج پخته | 1 عدد متوسط |  | |  | | |  | |  |  | |
| **69** | سير | 1 حبه |  | |  | | |  | |  |  | |
| **70** | پياز خام | 1 عدد کوچک |  | |  | | |  | |  |  | |
| **71** | پياز سرخ شده | 1قاشق غذاخوري |  | |  | | |  | |  |  | |
| **72** | انواع كلم | كاسه ماست‌خوري |  | |  | | |  | |  |  | |
| **73** | فلفل دلمه‌اي | 1 عدد متوسط |  | |  | | |  | |  |  | |
| **74** | اسفناج پخته | 1ليوان |  | |  | | |  | |  |  | |
| 75 | شلغم | 1 عدد متوسط |  | |  | | |  | |  |  | |
| 76 | فلفل سبز باريك | 1 عدد متوسط |  | |  | | |  | |  |  | |
| 77 | سس قرمز | 1قاشق غذاخوري |  | |  | | |  | |  |  | |
| 78 | ترشي | 1پياله |  | |  | | |  | |  | چند ماه سال؟ | |
| 79 | شور | 1پياله |  | |  | | |  | |  | چند ماه سال؟ | |
| 80 | خيار شور | 1 عدد متوسط |  | |  | | |  | |  |  | |
| 81 | طالبي و گرمک | 4/1 عدد |  | |  | | |  | |  |  | |
| 82 | خربزه | 1 قاچ متوسط |  | |  | | |  | |  |  | |
| 83 | هندوانه | 1 قاچ متوسط |  | |  | | |  | |  |  | |
| 84 | گلابي | 1 عدد متوسط |  | |  | | |  | |  |  | |
| 85 | زردآلو | 1 عدد متوسط |  | |  | | |  | |  |  | |
| 86 | گيلاس يا آلبالو | 1 پيش دستي کوچک |  | |  | | |  | |  |  | |
| 87 | سيب | 1 عدد متوسط |  | |  | | |  | |  |  | |
| 88 | هلو | 1 عدد متوسط |  |  | | |  | |  | |  | |
| 89 | شليل | 1 عدد متوسط |  |  | | |  | |  | |  | |
| 90 | گوجه سبز | 1 عدد متوسط |  |  | | |  | |  | |  | |
| 91 | انجير تازه | 1 عدد متوسط |  |  | | |  | |  | |  | |
| 92 | انجير خشك | 1 عدد متوسط |  |  | | |  | |  | |  | |
| 93 | انگور | 1 خوشه متوسط |  |  | | |  | |  | |  | |
| 94 | كيوي | 1 عدد متوسط |  |  | | |  | |  | |  | |
| 95 | گريپ فروت | 1 عدد متوسط |  |  | | |  | |  | |  | |
| 96 | پرتقال | 1 عدد متوسط |  |  | | |  | |  | |  | |

| **رديف** | **مقدار** | **مقدار غذايي** | **روز** | **هفته** | **ماه** | **سال** | **ملاحظات** |
| --- | --- | --- | --- | --- | --- | --- | --- |
| 97 | خرمالو | 1 عدد متوسط |  |  |  |  |  |
| 98 | نارنگي | 1 عدد متوسط |  |  |  |  |  |
| 99 | انار | 1 عدد متوسط |  |  |  |  |  |
| 100 | خرما | 1 عدد متوسط |  |  |  |  |  |
| 101 | آلو (زرد و قرمز) | 1 عدد متوسط |  |  |  |  |  |
| 102 | توت فرنگي | 1عدد |  |  |  |  |  |
| 103 | موز | 1 عدد متوسط |  |  |  |  |  |
| 104 | ليمو شيرين | 1 عدد متوسط |  |  |  |  |  |
| 105 | ليمو ترش | 1 عدد متوسط |  |  |  |  |  |
| 106 | آب پرتغال | 1 ليوان |  |  |  |  |  |
| 107 | آب سيب | 1 ليوان |  |  |  |  |  |
| 108 | آب طالبي | 1 ليوان |  |  |  |  |  |
| 109 | كشمش | 1قاشق غذاخوري |  |  |  |  |  |
| 110 | توت تازه | 1 پيش دستي |  |  |  |  |  |
| 111 | توت خشك | 1عدد |  |  |  |  |  |
| 112 | برگه ها | 1عدد |  |  |  |  |  |
| 113 | زيتون سبز | 1عدد |  |  |  |  |  |
| 114 | كمپوت ميوه‌جات | 1 قوطي |  |  |  |  |  |
| 115 | آبميوه بسته بندي شده | 1عدد |  |  |  |  |  |
| 116 | روغن نباتي جامد | 1قاشق غذاخوري |  |  |  |  |  |
| 117 | روغن مايع | 1قاشق غذا خوري |  |  |  |  |  |
| 118 | روغن زيتون | 1قاشق غذاخوري |  |  |  |  |  |
| 119 | روغن حيواني | 1قاشق غذاخوري |  |  |  |  |  |
| 120 | سس مايونز | 1قاشق غذاخوري |  |  |  |  |  |
| 121 | بادام زميني | 1عدد |  |  |  |  |  |
| 122 | بادام | 1عدد |  |  |  |  |  |
| 123 | گردو | 1 عدد مغز كامل |  |  |  |  |  |
| 124 | پسته | 1 عدد |  |  |  |  |  |
| 125 | فندق | 1 عدد |  |  |  |  |  |
| 126 | تخمه (هندوانه كدو,آفتابگردان) | كاسه ماست خوري |  |  |  |  |  |
| 127 | قند، شكرپنيريا نقل | 1 عدد |  |  |  |  |  |
| 128 | شكر | 1قاشق مربا خوري |  |  |  |  |  |
| 129 | عسل | 1قاشق مربا خوري |  |  |  |  |  |
| 130 | مربا (با نوع) | 1قاشق غذاخوري |  |  |  |  |  |

| **رديف** | **مواد غذايي** | | **مقدار** | **روز** | **هفته** | **ماه** | **سال** | **ملاحظات** |
| --- | --- | --- | --- | --- | --- | --- | --- | --- |
| 131 | نوشابه هاي کولادار يا صنعتي | | 1 ليوان |  |  |  |  |  |
| 132 | شيريني خشك | | 1 عدد متوسط |  |  |  |  |  |
| 133 | شيريني تر | | 1 عدد متوسط |  |  |  |  |  |
| 134 | گز | | 1 عدد متوسط |  |  |  |  | آردي * لقمه‌اي * |
| 135 | سوهان | | 1 قطعه |  |  |  |  |  |
| 136 | پفك | | 1 بسته |  |  |  |  |  |
| 137 | شكلات کاکايويي | | 1 عدد |  |  |  |  |  |
| 138 | چاي | | 1 ليوان |  |  |  |  |  |
| 139 | نمك | 1 قاشق چايخوري | |  |  |  |  | نوع:....... |
| 140 | چيپس | 1 بسته | |  |  |  |  |  |
| 141 | قهوه | 1 فنجان | |  |  |  |  |  |
| 142 | آبليمو | 1 قاشق مربا خوري | |  |  |  |  |  |
| 143 | نبات و آبنبات | 1 تكه متوسط | |  |  |  |  |  |
| 144 | قارچ پخته | نصف ليوان | |  |  |  |  |  |
| 145 | حلوا خانگي | 1قاشق غذاخوري | |  |  |  |  |  |
| 146 | حلوا شکري | 1قاشق غذاخوري | |  |  |  |  |  |
| 147 | انواع ادويه ها | 1قاشق چاي خوري | |  |  |  |  |  |

تاريخ تحويل پرسشنامه غذايي به ساکنين محترم: نام کارشناس تغذيه مسيول تکميل FFQ:

تاريخ تحويل به کارشناس تغذيه:
